# Malignant masquerade sclerosing mesenteritis: A case report and review

**DOI:** 10.1016/j.ijscr.2018.10.042

**Published:** 2018-11-03

**Authors:** Adithya G K, Satya Prakash Jindal, Varun Madaan, Rigved Gupta, Vivek Tandon, Deepak Govil

**Affiliations:** Indraprastha Apollo Hospital Delhi, India

**Keywords:** Mesenteric malignancy, Mesenteritis, Mesenteric fibrosis

## Abstract

Mesenteric panniculitis (MP), also known as sclerosing mesenteritis (SM), retractile mesenteritis or mesenteric lipodystrophy, is a rarely diagnosed inflammatory condition of unknown etiology that involves the mesenteric adipose tissue. We are presenting a case of sclerosing mesenteritis which presented us with features suggestive of malignant lesion and turned out to be benign one. An elderly gentleman of 68 years male presented to us with history of nonprogressive pain abdomen since 2 months associated with low grade fever and on and off constipation. On evaluation with CECT he was found to have mesenteric mass probably malignant. PET/CT showed an FDG avid lesion suggestive of malignancy, requiring surgery. He underwent radical excision of mesentery with resection of corresponding bowel. Final histopathology report showed poorly defined lesion comprised of nodular, poorly defined proliferation of bland looking fibroblast like cells, which at places are lying in fascicles, in a background of dense fibrosis and mature fat cells, with prominent focal lymphocytic infiltrate, at place forming reactive follicles, suggestive of sclerosing mesenteritis. Now patient is on follow up for further therapy.

## Highlights

• Sclerosing mesenteritis (SM) is difficult to be diagnosed preoperatively if careful attention is not given at imaging ordered.
• Vessel encasement seen on imaging is not definite till you confirm it during surgery.
• Debilitating disease even with complete excision.
• Long term follow up is required with good palliative care.

## 1 Introduction

Mesenteric panniculitis (MP), also known as sclerosing mesenteritis (SM), retractile mesenteritis or mesenteric lipodystrophy [1], is a rarely diagnosed inflammatory condition of unknown etiology that involves the mesenteric adipose tissue [2]. SM is histologically characterized by chronic nonspecific inflammation of the adipose tissue of the mesentery with large number of lipid-laden macrophages scattered among fat cells or fully replacing them. This usually represents a reaction to fat necrosis (may be due to trauma). The rarity of this condition has limited our ability to study demographic picture, clinical features, natural history and response to therapy. Therefore treatment decisions are guided by anecdotal experience, small case series or case reports. In that attempt we are adding one more case report in order to understand it better.

## 2 Case report

**Fig. 1:**
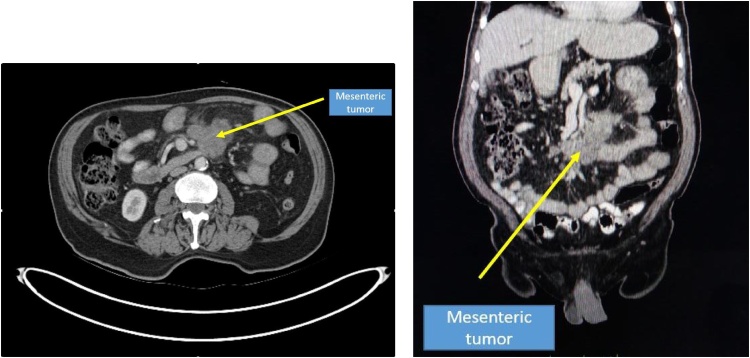
CECT abdomen showing large mesenteric mass, not seem to infiltrate surrounding structures.

**Fig. 2:**
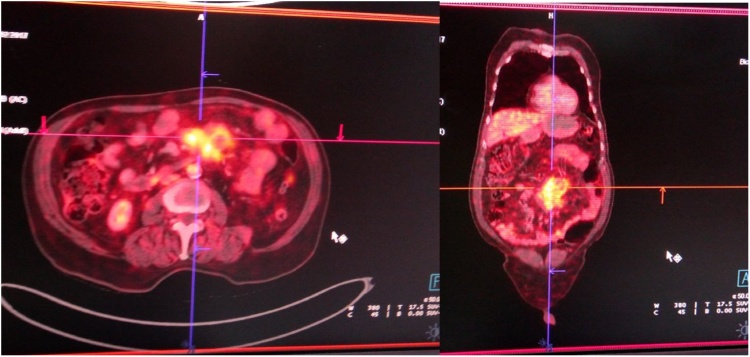
PET/CT scan showing FDG avid mass in the mesentery.

A 68 year old patient presented to us with history of nonprogressive pain abdomen since 2 months associated with low grade fever. Gives history of constipation on and off. No history of loss of appetite or loss of significant weight. Abdominal examination reveals a large slightly mobile, nontender mass in the left hypochondrial region. USG guided biopsy showed spindle cell mesenteric lesion with suspicion for GIST. Patient was evaluated with PET/CT abdomen which showed FDG avid lesion in mesentery measuring 5*6.4*8.7 cm, metabolically active perilesional and right supraclavicular lymph nodes suspicious of metastasis (Fig. 1, Fig. 2). In view of unsettled diagnosis, exploratory laparotomy was planned with probable resection of mesenteric lesion and excisional biopsy of right supraclavicular lymph nodes.

**Fig. 3:**
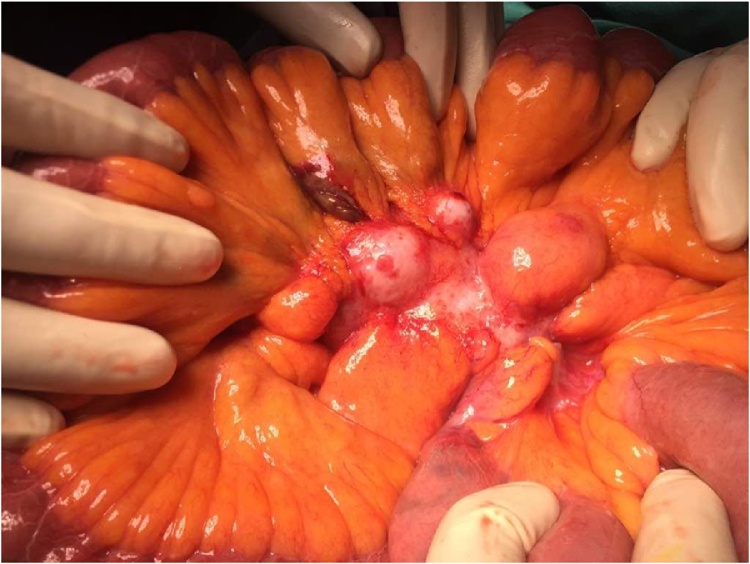
Intraoperative picture of mesenteric mass.

**Fig. 4:**
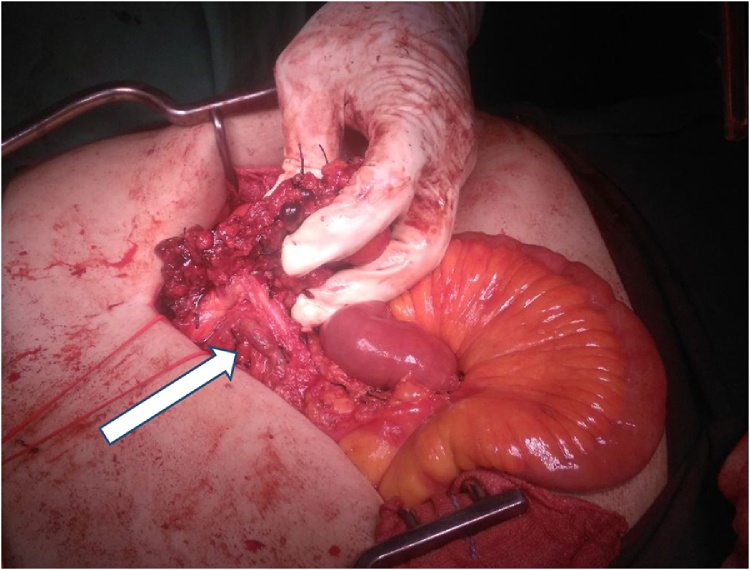
Superior mesenteric artery and vein being preserved after the surgery (arrow).

**Fig. 5:**
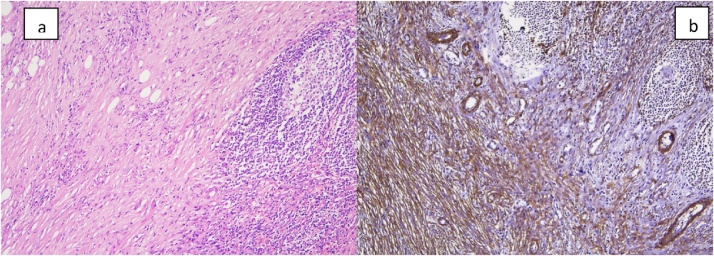
Histopathology slides: a) H&E stain – bland fibroblast like cells in sheets and fascicles, mature fat cells, lymphoplasmacytic infiltrate and reactive lymphoid follicles. b) IHC stain for SMA (smooth muscle actin): Highlights the myofibroblastic nature of the spindle cells.

Intraoperatively there was a large mesenteric tumor measuring about 10*8 cm abutting SMA and SMV extending till the root of duodenojejunal flexure (DJF). Middle colic artery was completely encased by the tumor. Bowel and peritoneum was healthy. Minimal chylous ascites was noted. Few enlarged right supraclavicular lymph nodes were excised for biopsy (Fig. 2, Fig. 3, Fig. 4). Excision of the tumor with bowel resection and anastomosis done. Patient recovered well and discharged on postop day 7. Final histopathology report showed mesenteric mass with poorly defined proliferation of bland looking fibroblast like cells, in a background of dense fibrosis and mature fat cells exhibiting features of sclerosing mesenteritis. Stain for Ckit and Alk-1 were negative. The mib-1 labelling index in the spindle cell areas is about 5%. Supraclavicular lymph node showed reactive lymphadenitis. In subsequent follow up patient is planned for medical treatment in the form of tamoxifen and steroids (Fig. 5).

## 3 Discussion

The prevalence of MP in one of the cohort is 0.58%, which is similar to data from Daskalogiannaki and Kipfer et al, but in contrast to Canyigit et al. and Coulier who reported a higher prevalence of 2.43% and 7.83%, respectively. [[3], [4], [5]] SM mostly occurs in mid to late adulthood, showing a male predominance [3]. When SM is present, frequent symptoms are fever, abdominal pain, nausea, diarrhea or fatigue, especially if retraction of mesentery leads to bowel obstruction or less commonly to mesenteric ischemia [6]. In our case patient had low grade evening rise of temperature suggesting more in favor of tuberculosis. Previous studies suggested that SM is associated with malignancy, but none of these studies have proven it yet.

When it comes to the diagnosis of SM imaging studies may not help us in a great way. But imaging helps in getting the tissue biopsy for diagnosis. SM is most commonly diagnosed incidentally by CT scan MRI and USG being alternatives. Mass lesion, absence of infiltration of neighboring structures, inhomogeneity, hypodense fatty halo surrounding blood vessels and nodes and hyperdense pseudocapsules are the criteria given by Coulier, may help to differentiate SM from lymphoma, carcinoid tumor, carcinomatosis, primary mesenteric mesothelioma, and mesenteric edema. [2] PET-CT can be helpful in differentiating mere SM from SM co-existing with neoplasia. In our case PET/CT did not derive us at the diagnosis, intern it led us to a false diagnosis. In a case series published by Salma Akram et al the diagnosis was established at laparotomy with biopsy in 65%, laparoscopy with biopsy in 25%, and CT-guided biopsy in 10% [7]. In our case CT guided biopsy misled us to a nonspecific neoplastic process.

Spontaneous resolution can occur. Corticosteroids and other anti-inflammatory and immunosuppressive agents are adjuncts. Operative indications are diagnosis dilemma as in our case and acute intestinal obstruction. Rarity of this condition also makes intraoperative findings confusing during the laparotomy for most of the surgeons who would directly take it as neoplastic and do a radical resection.

The treatment of this condition is controversial. One study reported the successful use of one and half year course of tapering azathioprine (1 mg/kg/day) and a three-year tapering course of prednisone (0.5 mg/kg/day) after initial surgery. [8] Tamoxifen is being tried in some of the studies.

## 4 Conclusions

Patients with intractable bowel obstruction or uncertainty in diagnosis may require surgery without having a diagnosis in hand. However, long-term follow-up studies are needed to substantiate any approach.

## Conflict of interest

No conflicts of interest.

## Funding source

No.

## Ethical approval

Ethical exemption is done by hospital. If any details required later on will furnish it.

## Consent

Written informed consent was obtained from the patient for publication of this case report and accompanying images. A copy of the written consent is available for review by the Editor-in-Chief of this journal on request.

## Author contribution

Adithya G. K^1^, Vachan Hukkeri^2^, Satya Prakash^3^, Vivek Tandon^4^, Deepak Govil^5^ 1,2,3 Resident in Dept of GI surgery, Apollo Hospital Delhi, India (Assistant surgeon, Data analysis and manuscript writing) 5, 4 Senior consultant, Dept of GI surgery, Apollo Hospital Delhi, India (Operating surgeon, data retrieval and manuscript analysis).

## Registration of research studies

Not a research article.

## Guarantor

Adithya G K.

## Provenance and peer review

Not commissioned, externally peer reviewed.
